# Severe aplastic anemia as a rare autoimmune complication of ankylosing spondylitis: A rare case report

**DOI:** 10.1097/MD.0000000000050536

**Published:** 2026-09-04

**Authors:** Rama Alkhen, Mahmoud Alhamadeh Alswij, Mais Musleh, Yara Mohsen, Qossay ALHusein

**Affiliations:** a Department of Hematology, AL-MOUWASAT University Hospital,Faculty of Medicine, Damascus, Syria; b Department of Internal Medicine, Fellowship of Hematology, AL-MOUWASAT University Hospital, Damascus, Syria.

**Keywords:** ankylosing spondylitis, aplastic anemia, rare

## Abstract

**Rationale:**

Aplastic anemia (AA) is a rare, life-threatening bone marrow failure syndrome, most often driven by immune-mediated destruction of hematopoietic stem and progenitor cells. Ankylosing spondylitis (AS) is a chronic inflammatory spondyloarthropathy characterized by aberrant T-cell activity and elevated pro-inflammatory cytokines. Although both diseases share immune dysregulation as a common pathogenic feature, their coexistence has not been previously documented in the literature.

**Patient concerns:**

A 42-year-old male with a known history of AS presented with recurrent mucocutaneous bleeding, severe fatigue, and progressive transfusion dependence.

**Diagnoses:**

Laboratory work-up revealed pancytopenia. Bone marrow biopsy showed marked hypocellularity (5%–10%), consistent with severe AA. Secondary etiologies were systematically excluded, confirming the diagnosis of severe AA in the setting of underlying AS.

**Interventions:**

The patient was started on immunosuppressive therapy comprising antithymocyte globulin, cyclosporine, and eltrombopag. Antithymocyte globulin was discontinued due to serum sickness, while cyclosporine and eltrombopag were maintained. For active AS, golimumab (an anti-tumor necrosis factor-alpha agent) was subsequently added.

**Outcomes:**

Over a follow-up period of 6 months, the patient achieved normalization of peripheral blood counts and complete remission of rheumatologic symptoms, with no major adverse events reported.

**Lessons:**

This case suggests a possible immunological link between AA and AS, given their shared pathways of T-cell-mediated cytotoxicity and cytokine activation. However, this single observation should be interpreted with caution and does not establish causation. It may nonetheless contribute to the emerging literature on potential pathophysiological overlaps between rheumatologic disorders and bone marrow failure syndromes.

**Key learning points**

- Severe aplastic anemia may represent a rare clinical association in patients with ankylosing spondylitis.
- Combined immunosuppressive therapy for aplastic anemia and biologic therapy for ankylosing spondylitis was associated with concurrent improvement of both hematologic and rheumatologic manifestations.
- The parallel clinical response raises the possibility of overlapping immune mechanisms.
- This case highlights the systemic immune nature of ankylosing spondylitis and the potential extension of inflammation beyond the musculoskeletal system.

## 1. Introduction

Aplastic anemia (AA) is an acquired bone marrow failure syndrome characterized by pancytopenia and markedly hypocellular marrow. Most cases result from immune-mediated destruction of driven by aberrant activation of cytotoxic T cells and overexpression of interferon-gamma (IFN-γ) and tumor necrosis factor-alpha (TNF-α).^[1]^ The consequent loss of hematopoietic precursors leads to severe anemia, increased susceptibility to infections, and bleeding tendencies.

Ankylosing spondylitis (AS) is a chronic inflammatory disorder within the spondyloarthritis spectrum, primarily affecting the sacroiliac joints and spine. It predominantly affects young adults and is strongly associated with the HLA-B27 antigen. The disease arises from dysregulated immune pathways involving TNF-α, IL-17, and IL-23 signaling, which promote both inflammation and aberrant bone remodeling.^[2]^

Recent insights indicate that AS is not confined to entheseal inflammation but also involves the bone marrow as an early site of immune activation and osteitis.^[3]^ This bone marrow–centric model highlights the potential convergence between AS and immune-mediated bone marrow failure syndromes, where dysregulated T-cell responses and pro-inflammatory cytokine signaling form a shared pathogenic pathway underlying both rheumatologic and hematologic autoimmunity.

Notably, the coexistence of AA and AS represents a rare and scarcely reported clinical association, underscoring the importance of this case in expanding current understanding of immune-mediated overlap syndromes.

## 2. Case presentation

A 42-year-old male presented to the Hematology unit with a several-month history of recurrent gum bleeding, easy bruising, fatigue, and low back pain, requiring multiple red blood cell and platelet transfusions. His past medical history included untreated AS, diagnosed 5 years earlier and confirmed by HLA-B27 positivity. The patient also had a history of subfertility and benign intracranial hypertension, previously treated with a ventriculoperitoneal shunt, with stable follow-up. Family history was significant for AS in his brother.

On physical examination, the patient was hemodynamically stable. He exhibited pallor, scattered petechiae over the lower limbs and chest, and characteristic spinal deformities including kyphosis and a stooped posture. Lumbar spine examination revealed restricted lateral and anterior flexion consistent with a flexion deformity.

No hepatosplenomegaly or lymphadenopathy was noted, and the remainder of the physical examination was unremarkable. The patient was not taking any medications, was a lifelong nonsmoker, and had recently migrated from Turkey to Syria 6 months prior. He worked as a tailor, was married, and had 1 child conceived via in vitro fertilization.

Initial laboratory investigations revealed pancytopenia with a White blood cell count of 1.6 × 10^9^/L, hemoglobin 6.4 g/dL, and platelet count 19 × 10^9^/L. Additional findings included an absolute reticulocyte count of 1.78 × 10^9^/L, erythrocyte sedimentation rate of 112 mm/h, creatinine 0.8 mg/dL, lactate dehydrogenase 436 U/L, albumin 3.2 g/dL, alanine aminotransferase 13 U/L, aspartate aminotransferase 19 U/L, calcium 8.5 mg/dL, triglycerides 46 mg/dL, prothrombin time 100%, International normalized ratio 1.0, partial thromboplastin time 34 seconds, serum iron 215 µg/dL, total iron-binding capacity 306 µg/dL, transferrin saturation 70.5%, thyroid-stimulating hormone 1.5 mIU/L, vitamin B12 312 pg/mL, and folate 7 ng/mL, antinuclear antibody negative.

Serologic testing for hepatitis B surface antigen, anti- Hepatitis C virus, Human immunodeficiency virus, Cytomegalovirus and Epstein–Barr virus was negative, and tuberculosis screening was unremarkable (Table 1).

**Table 1:** 

White blood cells	1600/ microl
Neutrophil	784/microl
Hemoglobin	6.4 g/dL
Hematocrit	18 %
Mean corpuscular volum	97 fL
Platelet	19,000/microl
Absolute reticulocyte count	1790/microl
Urea	
Creatinine	0.8 mg/dL
Ast	19 u/l
Alt	13 u/l
Alb	3.2 g/dL
Pt	100%
Inr	1
Ptt	34 sec
Esr	112 mm/h
Calcium	8.5 mg/dL
Tsh	1.5 mIu/l
Tg	46 mg/dL
HLA-B27	Positive
Antinuclear antibody test	Negative
Complement c3	1.26 g/l
Complement c4	0.263 g/l
Hepatitis B antigen	Negative
Anti hepatitis C virus	Negative
Hiv igm/ igg	Negative
Cmv igm/ igg	Negative
Ebv igm/ igg	Negative
Skin tuberculin test	Negative
Ferritin	1526.14 ng/mL
Iron	215 micrgram/dL
Transferrin saturation	70.3 %
Erythropoietin	1036.5 miu/mL
B9	7 ng/mL
B12	312 pg/mL

Alb = Albumin, ALT = Alanine aminotransferase, ANA = Antinuclear antibody, AST = Aspartate aminotransferase, CMV = Cytomegalovirus, EBV = Epstein–Barr virus, ESR = Erythrocyte sedimentation rate, HIV = Human immunodeficiency virus, HLA-B27 = Human leukocyte antigen B27, INR = International normalized ratio, PT = Prothrombin time, PTT = Partial thromboplastin time, TG = Triglycerides, TSH = Thyroid-stimulating hormone, WBC = White blood cells.

**Figure 1.**
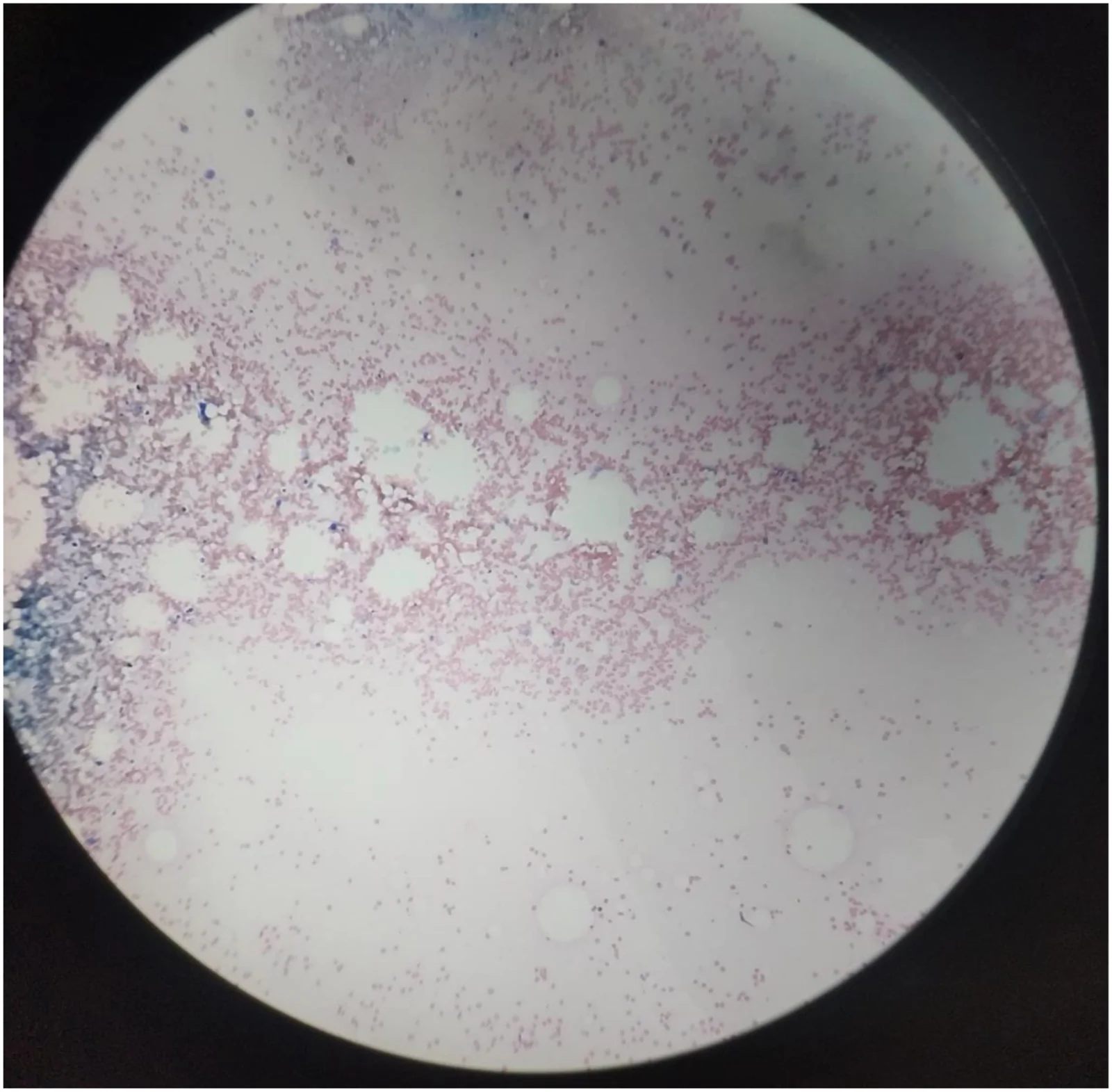
The bone marrow aspirate smear, stained with WrightGiemsa and examined under 10x magnification, is markedly hypocellular. Overall cellularity is severely decreased, estimated at 10% to 20%.

Cardiac evaluation by echocardiography was normal. Non-contrast computed tomography of the chest, abdomen, and pelvis showed no abnormalities. Peripheral blood smear demonstrated decreased cell counts with otherwise normal White blood cell and platelet morphology, while red blood cells exhibited marked hypochromasia. Bone marrow aspiration revealed markedly hypocellular marrow with 10% to 20% cellularity and no atypical cells (Fig. 1).

Bone marrow biopsy demonstrated a hypocellular marrow with 5% to 10% cellularity, prominent fatty replacement, and panmyeloid hypoplasia, consistent with AA The difference in cellularity between bone marrow aspiration (10%–20%) and trephine biopsy (5%–10%) likely reflects sampling variability. Trephine biopsy generally provides a more representative assessment of global marrow cellularity and, therefore, served as the primary reference for diagnostic classification. Flow cytometry showed no blast population, and paroxysmal nocturnal hemoglobinuria testing was negative. Cytogenetic analysis revealed a normal karyotype (46, XY). Based on these findings, the patient was diagnosed with AA.

**Figure 2.**
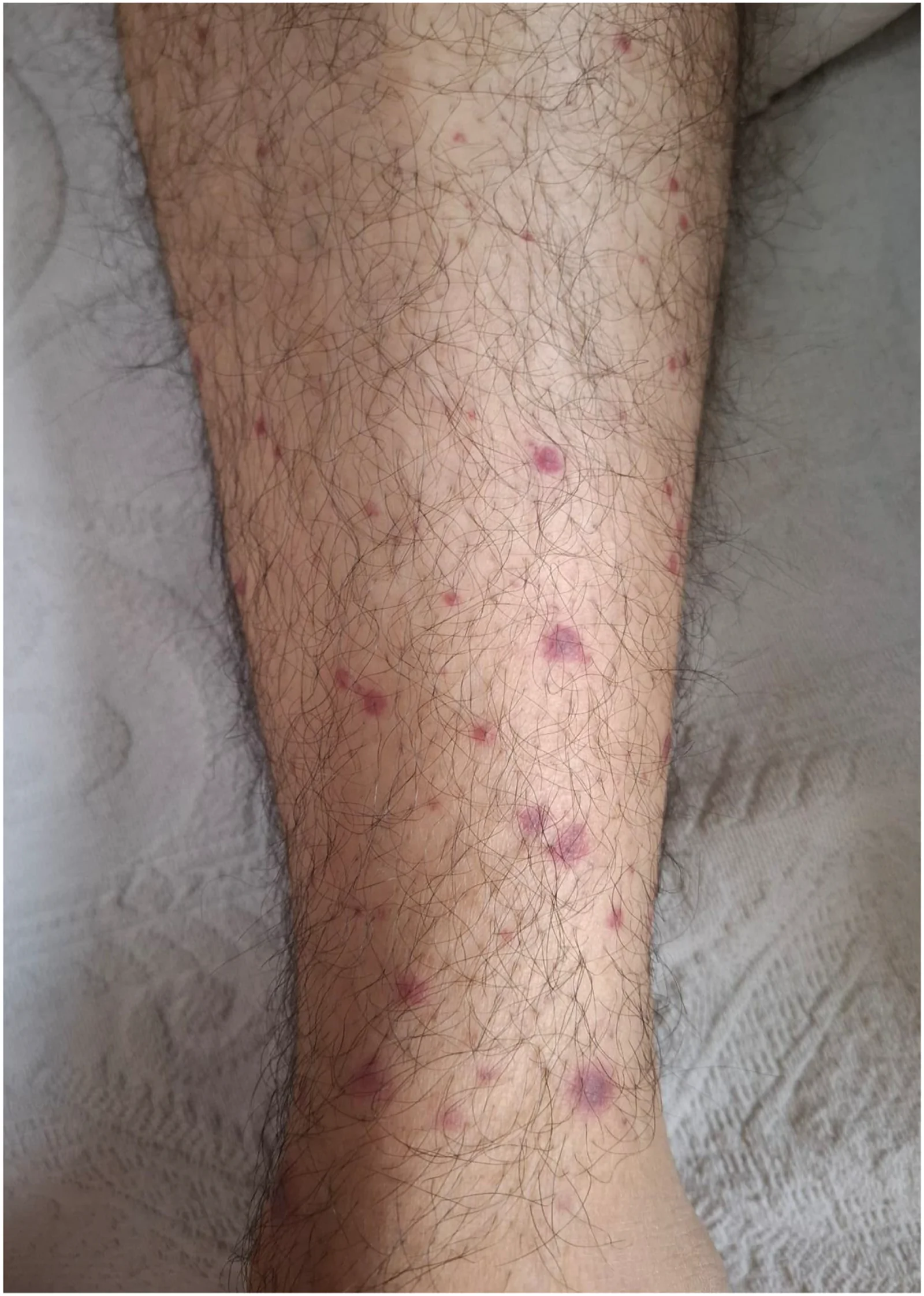
Palpable purpura on the lower limb, consistent with a serum sickness-like reaction following ATG therapy.

The patient initiated triple immunosuppressive therapy, including eltrombopag 150 mg orally once daily, oral cyclosporine 3 mg/kg twice daily, and rapid-derived antithymocyte globulin (ATG) 40 mg/kg IV over 4 hours. Premedication with chlorpheniramine 10 mg, paracetamol 1 g, and prednisolone 40 mg was administered to mitigate serum sickness. On day 2 of immunosuppressive therapy, he developed fever (39°C), rash, arthralgia, malaise, and palpable purpura (Fig. 2), suggestive of serum sickness. Infectious causes were excluded. ATG was discontinued, and intravenous methylprednisolone 1 mg/kg daily was initiated for 3 days, followed by oral prednisolone 1 mg/kg with gradual tapering, resulting in clinical improvement.

**Figure 3.**
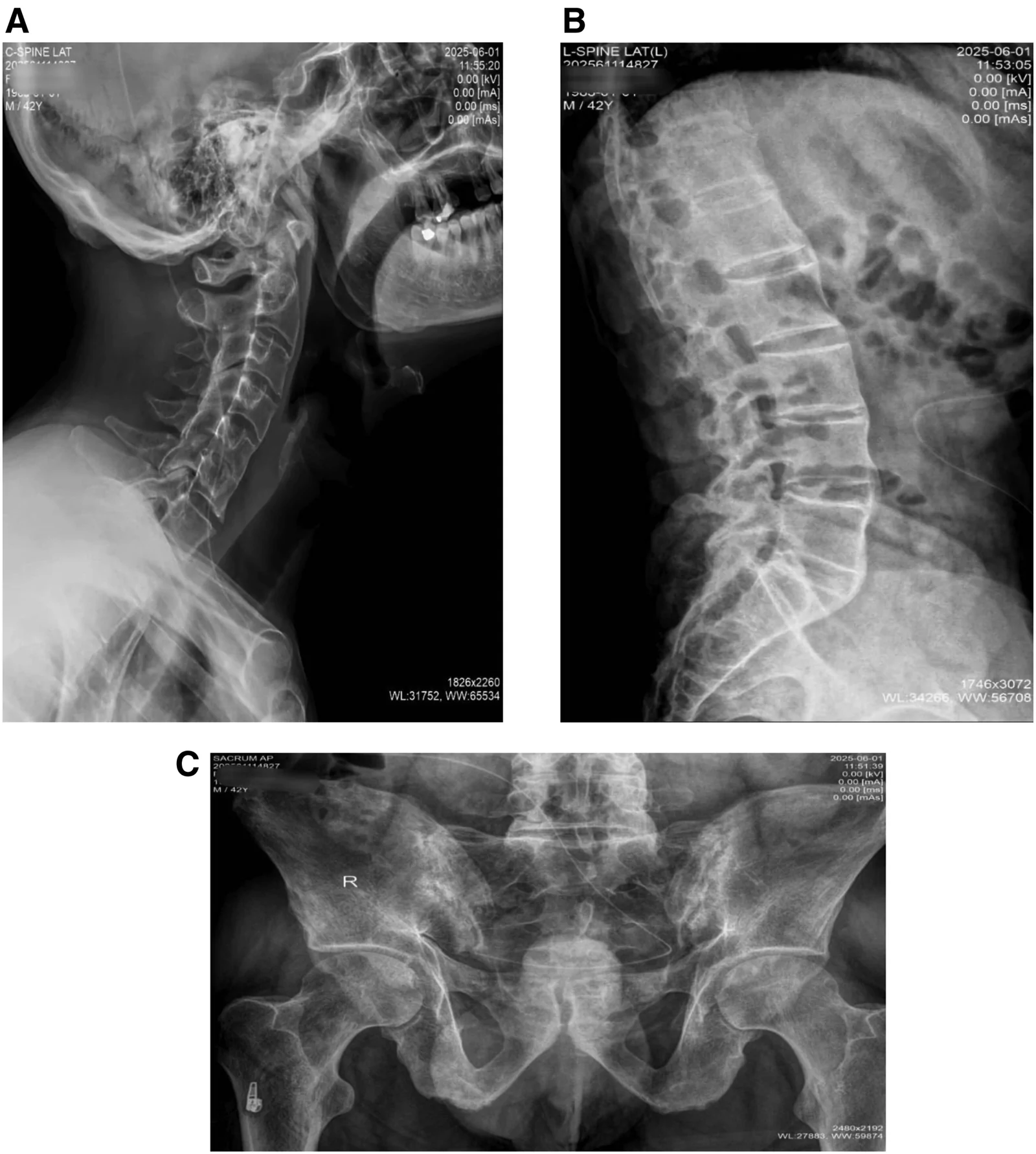
(A) C-Spine LAT Cervical spine radiograph demonstrates anterior ossification and early ‘bamboo spine appearance. (B) L-Spine LAT Lateral lumbar spine shows squaring of vertebral bodies and early syndesmophyte formation. (C) Sacrum AP: Bilateral sacroiliac joint erosions, sclerosis, and early intra-articular fusion.

Radiographs revealed cervical and lumbar syndesmophytes and grade 3 sacroiliitis on pelvic imaging (Fig. 3), while ophthalmic evaluation showed no uveitis. The patient received golimumab for management of AS. At 5-month follow-up, hematologic parameters had improved, with WBC 6.8 × 10^9^/L, hemoglobin 14.5 g/dL, and platelet count 224 × 10^9^/L (Table 2). A summary of the key hematologic parameters at presentation and follow-up is provided (Table 3).

**Table 2:** 

White blood cells	6800/ microl
Neutrophil	3800/microl
Hemoglobin	14.5 g/dL
Hematocrit	39.6 %
Mean corpuscular volum	112.3 fL
Platelet	22,000/microl

**Table 3:** Key hematologic parameters at diagnosis and follow-up.

Parameter	At diagnosis	5‑Month Follow-up
White blood cell count (×10^9^/L)	1.6	6.8
Hemoglobin (g/dL)	6.4	14.5
Platelet count (×10^9^/L)	19	224
Absolute reticulocyte count (×10^9^/L)	1.78	–
Erythrocyte sedimentation rate (mm/h)	112	–

## 3. Discussion

AA represents a form of bone marrow failure characterized by pancytopenia and markedly hypocellular marrow, with most cases resulting from immune-mediated destruction of hematopoietic stem and progenitor cells.

AS, in contrast, is a chronic inflammatory spondyloarthropathy primarily affecting the axial skeleton and driven by aberrant immune activation involving both innate and adaptive pathways. Although distinct in clinical presentation, both conditions involve immune dysregulation, particularly T cell–mediated cytotoxicity and overproduction of pro-inflammatory cytokines such as interferon-γ (IFN-γ) and tumor necrosis factor-α (TNF-α), which may represent partially overlapping immunological pathways.

Recent evidence has redefined AS not merely as an entheseal disorder but as a bone marrow–centric inflammatory disease. Imaging and histopathological studies demonstrate that bone marrow inflammation (osteitis) is an early and fundamental event in AS, often preceding enthesitis.^[2,3]^ This evolving understanding emphasizes the pivotal role of bone marrow immune activation in AS pathogenesis.

In our case, the patient presented with pancytopenia fulfilling the Camitta criteria for severe AA and bone marrow biopsy revealed marked hypocellularity (5%–10%) without abnormal infiltrates or fibrosis, in accordance with the British Society for Haematology guidelines.^[4]^ There was no evidence of secondary causes such as viral infections, myelotoxic drugs, or toxic exposures, directing attention instead toward the patient’s underlying autoimmune diathesis: active, treatment-naïve AS.

On review of the literature, no previous case has been reported describing the coexistence of severe AA and active AS in the same patient. Reports of other autoimmune hematologic manifestations in AS, such as pure red cell aplasia and immune thrombocytopenia, are exceedingly rare. Shao et al (2019) described a patient with inadequately controlled AS who developed pure red cell aplasia and achieved complete hematologic and rheumatologic remission following cyclosporine and corticosteroid therapy.^[5]^ Similarly, Bouchrane et al (2023) reported pancytopenia secondary to immune thrombocytopenia in AS, which improved with intensified immunosuppression.^[6]^ These observations collectively support the concept of shared immune mechanisms linking AS to bone marrow failure syndromes.

Potential overlap in the immunopathology of the 2 conditions may be interpreted in light of emerging immunobiological insights into AS. The same dysregulated immune pathways responsible for osteitis at entheseal sites may create a permissive environment for a generalized autoimmune assault on the bone marrow compartment. The anatomical communication between entheses and bone marrow through transcortical vessels could facilitate the spread of inflammatory mediators.^[2]^ Furthermore, mesenchymal stem cells (MSCs) in AS exhibit functional abnormalities such as aberrant cytokine production and heightened responsiveness to inflammatory stimuli that may promote activation and clonal expansion of cytotoxic T cells targeting hematopoietic stem and progenitor cells.^[2,7]^ Consequently, the development of severe AA in our patient could represent a possible manifestation of systemic immune dysregulation occurring in the setting of active AS, wherein autoreactive T cells cross-react with antigens in the hematopoietic niche. This mechanism parallels the established pathogenesis of idiopathic AA, in which activated T cells mediate HSPC destruction via Interferon gamma and tumor necrosis factor alpha signaling.^[1]^

The patient was treated with an immunosuppressive regimen including rabbit ATG, cyclosporine, and eltrombopag. ATG was discontinued early due to a serum sickness–like reaction, after which the patient continued cyclosporine and eltrombopag. Golimumab was later introduced to control AS activity, consistent with current international recommendations supporting the use of tumor necrosis factor alpha inhibitors in patients with active AS who have persistent disease activity despite adequate trials of nonsteroidal anti-inflammatory drugs, as outlined in the treatment guidelines of the American College of Rheumatology, Spondylitis Association of America, and SPARTAN.^[8]^ This modified regimen led to progressive hematologic recovery and marked clinical improvement over 6 months, culminating in normalization of blood counts and remission of AS symptoms.

The parallel resolution of pancytopenia and spondyloarthritic manifestations anti-following combined immunosuppressive and cytokine therapy raises the possibility of overlapping immune mechanisms. However, this temporal association should be interpreted cautiously, as a causal relationship cannot be established from a single case. Further studies are required to determine whether this observation reflects a true pathogenic link or a coincidental coexistence.

## Author contributions

**Data curation:** Rama Alkhen, Mahmoud Alhamadeh Alswij, Yara Mohsen.

**Formal analysis:** Rama Alkhen, Mahmoud Alhamadeh Alswij, Mais Musleh, Yara Mohsen.

**Investigation:** Rama Alkhen.

**Methodology:** Rama Alkhen.

**Resources:** Rama Alkhen.

**Software:** Mahmoud Alhamadeh Alswij.

**Project administration:** Mais Musleh.

**Supervision:** Qossay ALHusein.

**Writing – original draft:** Rama Alkhen, Mahmoud Alhamadeh Alswij, Yara Mohsen.

**Writing – review & editing:** Rama Alkhen, Mahmoud Alhamadeh Alswij, Mais Musleh, Qossay ALHusein.
